# Blockchain based electronic educational document management with role-based access control using machine learning model

**DOI:** 10.1038/s41598-025-99683-5

**Published:** 2025-05-29

**Authors:** P. Chinnasamy, B. Subashini, Ramesh Kumar Ayyasamy, Ajmeera Kiran, Binay Kumar Pandey, Digvijay Pandey, Mesfin Esayas Lelisho

**Affiliations:** 1 Department of Computer Science and Engineering, School of Computing, Kalasalingam Academy of Research and Education, Srivilliputtur, India; 2 Department of Data Science and Business Systems, School of Computing, SRMIST, Kattankulathur, Chennai, India; 3 Faculty of Information and Communication Technology, Universiti Tunku Abdul Rahman, Kampar, Perak Malaysia; 4 Department of Computer Science and Engineering, MLR Institute of Technology, Hyderabad, India; 5 Department of Information Technology, College of Technology, Govind Ballabh Pant University of Agriculture and Technology Pantnagar, Uttrarakhand, India; 6 Department of Technical Education (Government of Uttar Pradesh), Kanpur, Uttar Pradesh India; 7 Department of Statistics, College of Natural and Computational Science, Mizan-Tepi University, Tepi, Ethiopia

**Keywords:** Electronic educational document management, Blockchain, Role-based access, Temporal neural network, Remora swarm optimization, Computational models, Data processing, Machine learning, Software

## Abstract

The emergence of digital technology has led to a significant increase in the importance of educational credential storage, exchange, and verification for organisations, enterprises, and universities. Academic record forgery, record misuse, credential data tampering, time-consuming verification procedures, ownership and control difficulties, and other problems plague the education sector. Machine learning (ML) and blockchain, two of the most disruptive methods, have replaced traditional techniques in the education sector with highly technological and efficient ways. Our study aims to propose a novel electronic educational document management technique using a blockchain-based fuzzy feed-forward convolutional temporal neural network that detects malicious users. Here, the training is carried out based on NLP analysis in document word weight indexing. This document management access control is based on role-based access with simulated remora swarm optimisation. In order to identify malicious users, this suggested system logs access requests on the blockchain and authenticated users. The findings demonstrate that this suggested architecture performs as intended in every case. The experimental analysis is based on a malicious user detection dataset regarding Prediction accuracy, Mean average precision, F-measure, Latency, QoS, Contract execution time, and Throughput. Based on dataset feature analysis, the proposed B-FCTNN_SRSO achieved a prediction accuracy of 98%, a mean average precision (MAP) of 95%, and an F1 score of 97%, with a latency of 96%. Additionally, based on blockchain security analysis, the B-FCTNN_SRSO attained a QoS of 97%, a precision of 94%, and a throughput of 96%.

## Introduction

Systems for managing electronic documents are employed in many different sectors. Specifically, electronic document management systems are essential for streamlining government agency paperwork procedures and civil servants’ work by enabling easy, efficient access to documents. These systems also automate repetitive tasks like tracking down relevant information, searching for it, and creating reports on the flow of documents. Nonetheless, public authorities process a vast number of documents annually within set processing times, and the efficacy and efficiency of public authorities are primarily dependent on the calibre and productivity of document exchanges^1^. The daily requests may increase to several thousand as e-government advances. Intelligent algorithms will be more successful in government structures because documents and processes are stereotyped instead of systems with intricate and distinct organisational frameworks. In addition to preventing human error, machine learning may expedite processing of documents and prepare all data required for human decision-making. The filing cabinet’s development at the close of the nineteenth century marked the beginning of document management history. Edwin Granville Seibels created a vertical filing method in 1898 that arranges paper documents into boxes kept inside folded cabinets. For most of the 20 th century, these cabinets would remain dominant means of document storage in corporate sector^2^. A collection of completed documents that users have downloaded, such as books, dissertations, conference papers, newspapers, full-text journals, and other database contents, is known as educational resource data. Many materials are gathered in commercial databases with permission to use for data expression and intellectual property protection in educational resources; resources from libraries are also searched, downloaded, integrated into specific databases^3^. These resources can also be downloaded, copied, distributed, and have other features that have led to problems with intellectual property. ML educational resource data may be categorised into four categories based on various types of educational resources: corporate, personal, government, and other public institutions. Due to data transfer barriers, educational resources may lose control of educational secret data in an ML environment, even though educational secret data is protected by a data backup system^4^. Artificial intelligence (AI), encompassing ML and DL, is widely seen as a game-changer in numerous industries and sectors, including manufacturing, advertising, healthcare, telecommunication^5,38^, construction, and transportation. Since AI enables students to approach learning obstacles in a way that is customised to their unique experiences and interests, it will play a bigger role in higher education. AI-based digital learning techniques may adapt to each student’s knowledge level, preferred learning style, and learning objectives to help them get the most out of their education. In order to identify students’ areas of weakness and suggest courses that will improve their customised learning experience, it can also look at their prior academic records. In addition, teachers in higher education can devote more of their time to teaching and research by using AI to cut down on the time required for regular administrative activities^6^.

**Research Contribution of the Work**
1. To introduce a novel blockchain-based fuzzy feed-forward convolutional temporal neural network (B-FCTNN_SRSO) for secure educational document management and malicious user detection.
2. To implement role-based access control using simulated remora swarm optimization (SRSO) for efficient document access management.
3. To use natural language processing (NLP) for document word weight indexing to improve document management efficiency.
4. To evaluate the proposed system using a malicious user detection dataset based on various performance metrics such as: Prediction accuracy: 98%, Mean Average Precision (MAP): 95%, F1-Score: 97%, Latency: 96%, Quality of Service (QoS): 97%, Precision: 94%, Throughput: 96%.

## Related works

While some studies have attempted to predict student achievement, others have also classified educational data. Unal et al.^7^, focused on two sides of undergraduate students’ performance utilising DM approaches. The first step is to predict pupils’ academic performance at the conclusion of a four-year study programme. The second involves looking at how kids are developing and combining that with the results of predictions. He split the pupils into groups based on their levels of achievement: low achievement and high achievement. According to his findings, teachers must concentrate on a select group of courses that show especially strong or weak performance to provide timely warnings, assist underachievers, and provide guidance and opportunities for high achievers. Zhou & Huang^8^ used sixteen demographic variables, including age, gender, number of courses taken, internet connection, computer ownership, and attendance in class, to predict students’ academic success^9^. Among the ML methods, random forest, logistic regression, k-nearest neighbours, and SVM were able to predict students’ performance with prediction accuracy ranging from 50 to 81%. Heidari et al.^10^, created a model based on the students’ demographics and the grades they received for their in-term activities. In that study, classification methods based on Gradient Boosting Machine (GBM) were utilised to predict students’ academic progress. Findings indicated that nonattendance and achievement scores from the prior year were the best factors to use when estimating achievement scores. The authors discovered that demographic details like age, school, and neighbourhood may also be used to predict success or failure. The author presents a preliminary study about the creation, application, and delivery of LMS^11^. An overview of learning analytics is given in the paper to help combine learning with data. According to study’s findings, learning analytical methods are most prominent methods in the literature. The four processes involved in creating such models are gathering relevant data, reporting, forecasting, acting, and fine-tuning the learning environment in response to the data. This study does not cover specific machine-learning algorithms that perform well with the model. Likewise, Dewangan & Chandrakar^12^ summarises educational data mining by reviewing this area’s main ideas. Both studies summarised and explained the existing learning analytics and the subject of educational data mining and its methods, deviating from the systematic literature review requirements.

In addition, Wu^13^ offered an overview of educational data mining in another thoughtful literature review study. Rajendran et al.^14^ used input data like gender, wealth, board marks, and attendance to forecast students’ performances utilising ML algorithms like C4.5, sequential minimum optimisation (SMO), Naïve Bayes, 1-NN (1-Nearest Neighbourhood), MLP (multi-layer perceptron). After implementing correlation-based feature selection (CBFS) strategies to enhance method performance, they discovered that SMO outperforms other approaches in terms of effective average testing prediction accuracy, coming in at 66%. The author used artificial neural networks (ANNs)^15,55^ to forecast student performance. When these methods were used with input characteristics, including grades, study periods, and test scores, they attained a high prediction accuracy of 85%. Razak et al., identified at-risk students before the next course, the author employed logistic regression, SVMs, decision trees (DTs), artificial neural networks (ANNs), NB classifier (NBC)^16^. Input components from an offline course were employed in this study, including grades, attendance, quizzes, weekly homework, team participation, project milestones, mathematical modelling activities, and exams. According to an analysis of data, NBC algorithm produced predictions with acceptable accuracy (85%). A study by Zhang^17^ employed ML methods to forecast students’ academic achievement in engineering courses. Exam results were the study’s output variable, and course grades from every semester were among the input features. The researchers found that while multilinear regression is useful for predicting success of every student in a course, support vector machines (SVMs) are better suited for predicting a single student’s performance. Rajendran et al.^18^, researched the most effective classifier to use social and personal input variables to predict students’ success in higher education. Through analysis of logs generated while students were using computers, certain probabilistic models—such as Bayesian knowledge tracing—have been utilised to forecast students’ performance. These models, however, are unable to forecast pupils’ latent tendencies. In a comparable setting, Navimipour et al.^19^, developed a hybrid adaption system that groups students according to commonalities and suggests the best learning materials for each group. To construct learner profiles, this system considers the users’ past activities, learning preferences, and knowledge levels. Next, the Nearest Neighbour algorithm (KNN) is used to group learners. As a result, it offers adjustments based on the characteristics of the acquired learner group rather than on an individual basis. Fahd et al.^20^, used reinforcement learning-based adaption approach. All that this system needs to adjust and recommend is a learning path to meet the demands of the learners, which is their learning style. Similarly, Shi et al.^21^, suggested an adaptive e-learning method architecture based on reinforcement learning and a multi-agent system technique to suggest an adaptive learning path for a student who fits the following profile: verbal learning style, hearing impairment, and intermediate knowledge level.

## Proposed model

**Fig. 1:**
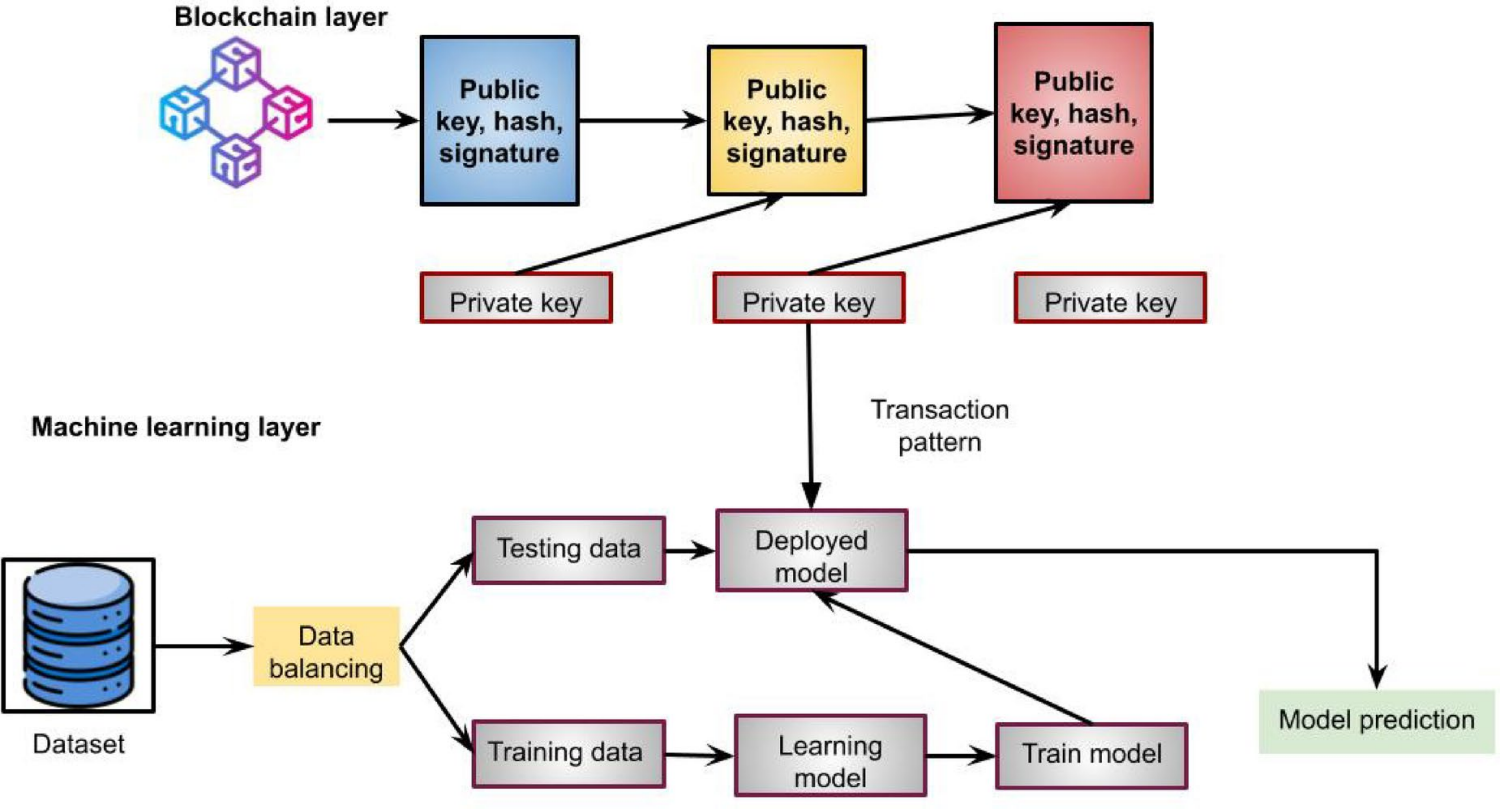
Proposed blockchain based machine learning model.

In addition to structured data, there is a significant amount of unstructured and semi-structured data related to educational resources. Structured data is defined as having a set format and being of a specific length. Data without a set format and variable length are called unstructured data. Early on, copyright was typically used to protect data expression and products of machine learning-based educational resources. However, copyright only covers expressing ideas and data, not the data itself. It also covered the work’s selection, arrangement, system, and structure. Additionally, the data are made available for public dissemination. In that case, a more significant number of persons will unavoidably come into contact with them, and the service provider cannot ensure that the data’s intended use will adhere to legal requirements, hence raising the possibility of copyright infringement. In this scenario, the user and data operator enter into a legally binding agreement through a licence agreement for data work about purchasing a copyright licence for data work. The proposed blockchain-based machine learning model in data analysis is shown in Fig. 1.

**Data Preparation**
- This data set undergoes data cleaning to minimise noise and missing values. After removing the missing records, the data set is reduced to 133. Data set originally contained 17 missing values in various aspects from 150 records. There are 48 females and 85 males in the data set. The stage ID consists of 22 high level, 47 middle level, and 64 lower level. In addition, students are divided into three sections: section A has 69 students, section B has 49 kids, and section C has 15 students. One hundred eleven students have their father as their contact person, and 22 students have their mother as their contact person.

## NLP analysis in document word weight indexing

**Fig. 2:**
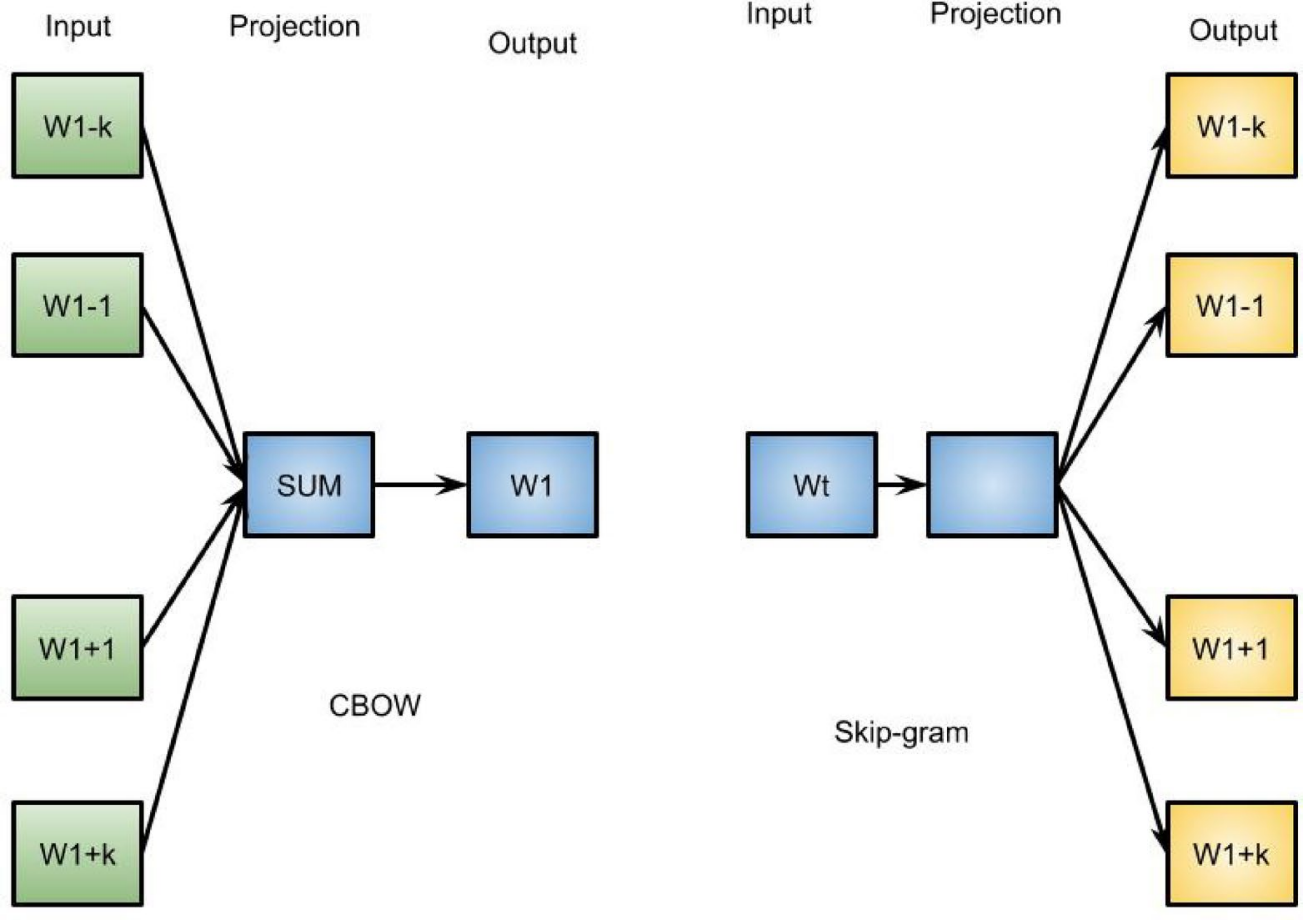
Continuous Bag-of-Words and skip-gram methods.

For word embedding models, we use the word2vec framework for training. The framework implements two distinct models and training methodologies. The first method, the Continuous Bag-Of-Words (CBOW) technique, attempts to anticipate a word by using its context—the surrounding words—as input. The other approach, the skip-gram method, guesses a word’s context using the word itself as input. Figure 2 shows a graphical representation of both approaches, with t representing the current word’s location and k representing the context window’s size. While the skip-gram approach works better for infrequent words, the CBOW model is faster overall. CBOW is less accurate in predicting unusual words because it averages the context word vectors to forecast the centre word. We have two sentences: “The food was devine” and “The food was delicious.” CBOW predicts words of interest based on context. Now that we want to predict the final word in the context [the food, was], the model is far more likely to suggest “delicious” because CBOW predicts the most likely word.

The word embedding algorithm is first algorithm. It is preferable to use this approach for rare words. The negative sampling algorithm is second training algorithm. Framework contains a large number of parameters. Consider simply the most important parameters. First, the dimensionality parameter finds number of dimensions of word vectors; generally speaking, a greater dimensionality is preferable. Nevertheless, the computing time increases with the number of dimensions. Next, depending on how close a word is to another word, the word2vec context window size parameter calculates the number of words that make up the word’s context. Moreover, minimum word frequency parameter determines how frequently a word appears in corpus to be considered. CBOW architecture trains method, centre word vector is found by taking the mean of the context vectors. Lastly, the noise word parameter is set to three and negative sampling is employed.

**Figure:**
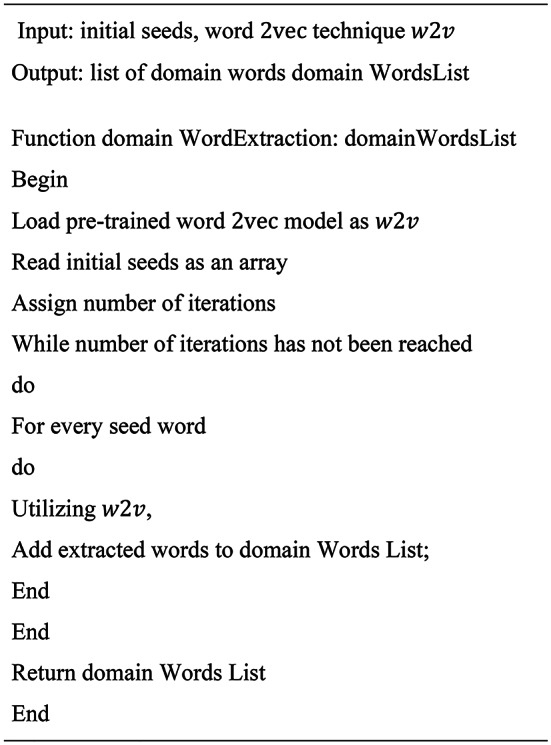
**Algorithm 1**Domain Words Extraction

search surface g (x, y, z) and template f (x, y, z) are matched using least squares. In the perfect world, one would have by Eq. (1)

1 $$f (x, y,z) = g(x, y,z)$$

Random errors have an influence, Eq. (2) is inconsistent.

2 $${\rm f(x,y,z)\:-\:e(x,y,z)\:=\: g(x,y,z)}$$

3 $$\:f:={\left[\begin{array}{llll}1&\:{f}_{1}&\:\cdots\:&\:{f}_{n}\end{array}\right]}^{\text{top}}\in\:{\mathbb{R}}^{n+1},l:={\left[\begin{array}{lll}{l}_{1}&\:\cdots\:&\:{l}_{n}\end{array}\right]}^{\text{top}}\in\:{\mathbb{R}}^{n}$$

Consider that by Eq. (4)

4 $$\:G(q,\theta\:):=\frac{L(q,\theta\:)}{F(q,\theta\:)}$$

Impulse response coefficients $\:\:\left\{{g}_{k}\left(\theta\:\right)\right\}$based on Eq. (5)

5 $$\:G(q,\theta\:)=\sum\:_{k=1}^{{\infty\:}}\:\:{g}_{k}\left(\theta\:\right){q}^{-k}$$

and suppose that the impulse response, which we represent by {gˆk}, has yielded the first N coefficients {gk(θ)} that we have measured by Eq. (6)

6 $$\:{\epsilon\:}_{k}\left(\theta\:\right)={\stackrel{\prime }{g}}_{k}-{g}_{k}\left(\theta\:\right)$$

By minimising the error criterion, we can derive an estimate of θ by Eq. (7)

7 $$\:{V}_{N}\left(\theta\:\right)={\sum\:}_{k=1}^{N}\:{e}_{k}^{2}(f,l)$$

The following can be used to accomplish the minimising in place of doing it directly. Take note that by revising by Eq. (8)

8 $$\:F(q,\theta\:)G(q,\theta\:)-L(q,\theta\:)=0$$

and enlarging its polynomials, we are able to express the relationship between each coefficient as Eq. (9)

$$\:\left[\begin{array}{l}l\\\:0\end{array}\right]=\left[\begin{array}{l}{Q}_{1}\\\:{Q}_{2}\end{array}\right]f$$

9 $$\:{Q}_{4}=\left[\begin{array}{ccccc}{g}_{2}&\:0&\:\cdots\:&\:0&\:0\\\:{g}_{2}&\:{g}_{1}&\:\cdots\:&\:0&\:0\\\:\vdots&\:\vdots&\:\vdots &\:\vdots&\:\vdots \\\:{g}_{n}&\:{g}_{n-1}&\:\cdots\:&\:{g}_{1}&\:0\end{array}\right]\in\:{\mathbb{R}}^{n+(n+1)}$$

### Electronic educational document management using blockchain based fuzzy Feed-Forward convolutional Temporal neural network (B-FFCTNN)

**Fig. 3:**
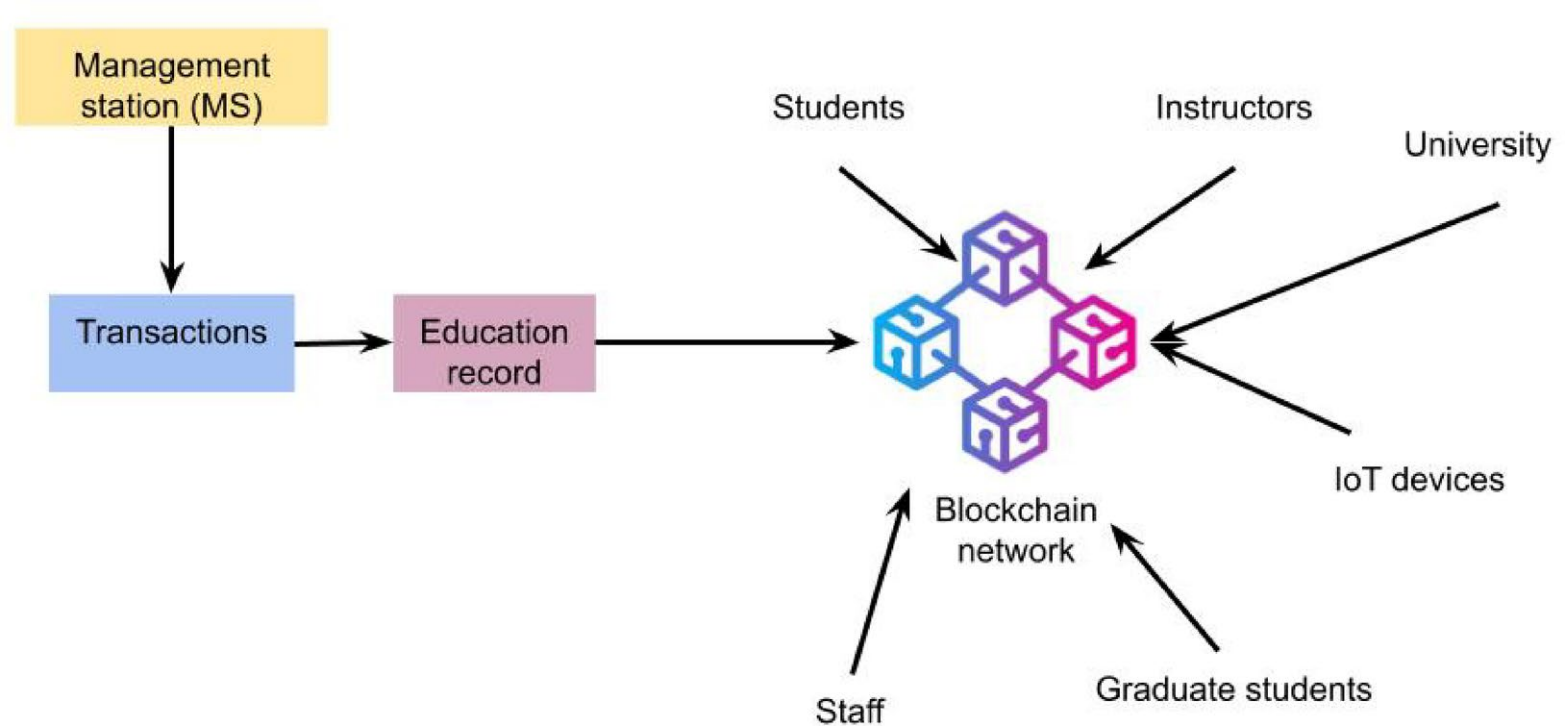
User interactions at each university within the blockchain network.

An overview of user interactions inside the blockchain network at each university is presented in Fig. 3. We assume all user devices in this proposed architecture have limited power, memory, and computing capabilities to communicate with the blockchain. Unregistered or unverified user devices cannot authenticate and, as a result, are prohibited from communicating with authorised devices within or outside of the same university. Following this process decreases the likelihood of a malicious device connecting with a legitimate device. Authorised university staff members have public access to all network records and can validate the academic records of any linked university’s students in the blockchain. For example, because their academic information is accessible to all relevant university officials regardless of location, students can enrol in courses, and instructors can apply to teach courses at other connected institutions within the network via the blockchain.

Additionally, the system lets employers or other educational institutions confirm the validity and integrity of the certificates that graduating students receive by allowing them to be issued on the blockchain. Records are made for these transactions in order to confirm the execution of the activities and validate the transactions. Subsequently, the learning transaction records are dispersed throughout the blockchain network to offer distributed authentication and authorisation to users and their registered devices.

The blockchain-based access control for student academic records is intended to give students authority over who can view their personal health data while offering an effective and safe way to manage their academic records. This architecture creates a decentralised system that guarantees data privacy and protection by utilising the Ethereum blockchain and solidity intelligent contract language. Smart contracts are used in this process to provide and cancel access authorisation. Smart contracts allow for coding interaction rules between entities, which are then automatically carried out when triggered. For the desired task, the contract has three primary mappings: authorizedUsers(), accessPermissions (), and record(). An authorised user list for access to student educational records can be found in the authorizedUsers() mapping. A list of students’ educational records that every user is permitted to access is contained in accessPermission() mapping. The record() mapping, as illustrated in Fig. 3, has a list of students’ academic records hashed on the blockchain. In contrast, contract owners have an even workload to validate each transaction that subjects seek.

**Fig. 4:**
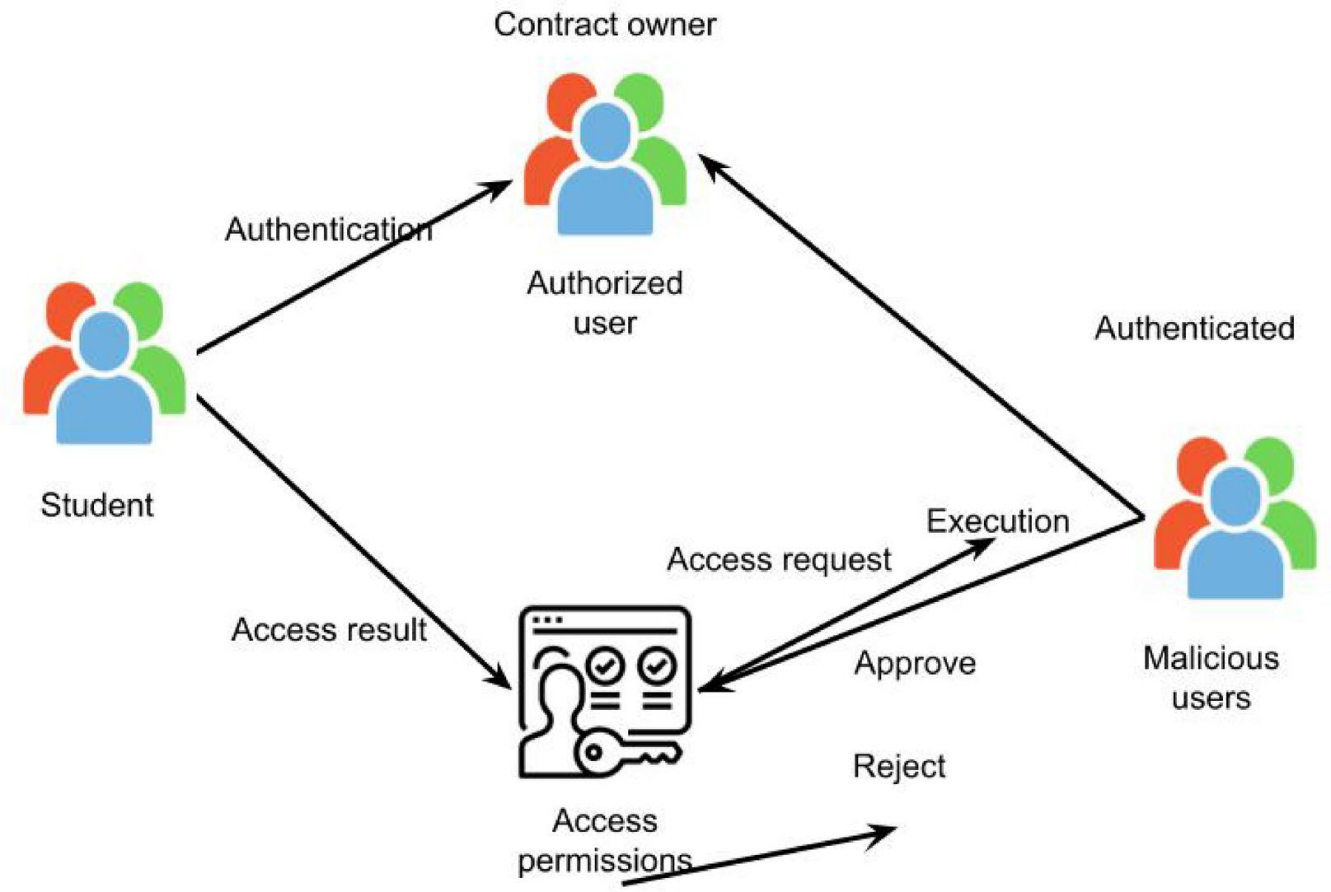
Accessing records utilizing smart contracts.

The address of student, hash of their academic record, a timestamp, description of transactions are all stored in Record struct. Following is a description of the mappings that were used to implement the smart contract:

- authorizedUsers(): an address to boolean mapping. List of authorised users is stored there. This function verifies the presence of the student record. Function notifies other users on network that a user’s access to a student’s educational record is cancelled if record is not present. In event that record is found, a new item is made in authorised user’s mapping with authorised user’s address and a Boolean value designating whether or not user given access to record.
- accessPermissions(): a mapping from addresses to booleans via a hierarchical mapping of record hashes. Every user’s and record’s access permissions are stored there. The allowAccess() function sets levels of access authorization for authorised user to true and verifies that student using function is owner of student’s educational record. RevokeAccess() can be triggered if user access permission is set to false.
- record(): uses addrecord, updaterecord, and getrecord, to perform new addition, update, obtain details. A student runs the addRecord() function to add a new student’s educational record to blockchain. If there isn’t already a record of the students, the method’s goal is to make one. UpdateRecord() function modifies record’s description and verifies that students calling it are ones who hold educational record. An authorised user must use getRecord() with their address and hash of student’s educational record in order to obtain the student’s record. The getRecord function in Fig. 4 retrieves the student’s educational record if access is allowed to the authorised user after verifying that access has been permitted.

**Fig. 5:**
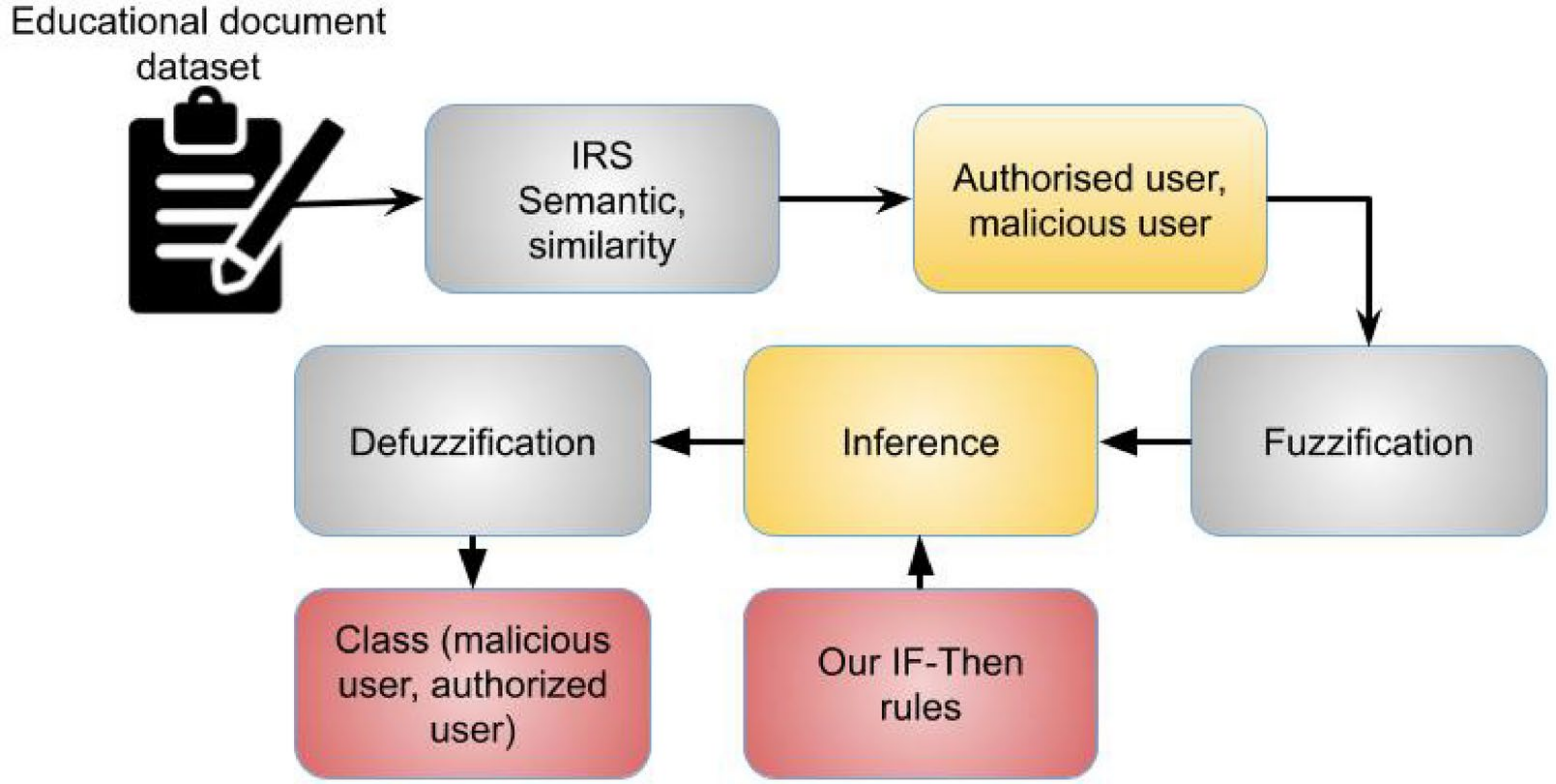
Fuzzy based malicious user detection.

In order to verify blocks and update blocks in the contract, parallel execution mode is used. Because the block formation and verification processes are carried out in a concurrent execution mode, the network’s contract execution performance also reduces time consumption. Every contract in the block is carried out sequentially by the miner. Control can move from one contract’s code to another contract’s code and back again when one contract calls upon the features of another contract. Concurrent transaction validation is another option. Miners’ proposed transactions may be re-executed by validators in a different order, producing an unanticipated result in block rejection. The two measures from Fig. 5 that are fed into fuzzy logic system are based on semantic similarity between student records and two opinion texts. Tweet’s class is its output. As previously said, defining inputs and outputs—definition of linguistic variables in input and output for our proposed FLS—is the first stage in an FLS.

In this instance, we define two input variables—positivity and negativity of educational data users—and one output variable—class of tweets—because we wish to categorise users of educational data into three classes: positive, negative, neutral. Any variable in an FLS, whether input or output, is called a linguistic variable. Linguistic terms, or fuzzy sets, are the values that any linguistic variable can have. Any variable in an FLS, whether input or output, is called a linguistic variable. Linguistic terms, or fuzzy sets, are the values that any linguistic variable can have. Determining crisp values of inputs that start our method is the next stage in our FLS after we have described linguistic variables as well as their linguistic words in input and output. 50% of the dataset was utilised to train the model, which is then used to map words onto their corresponding vector representations. Softmax probability is computed for each word to determine high-dimensional vectors for each word. Dimension of vector is associated with the quantity of neurons in buried layer. Vector dimension of each word starts at 100. To ensure that each sentence’s length is consistent across the dataset, zero vectors pad it. After that, a sentence vector X = {w1, w2,…,wi,…, w|x|} is constructed for each review x, X ∈ Rd×|x|, where wi denotes word embedding at associated position i in a sentence. The convolutional neural network is then fed X.

Convolution Layer: This layer encompasses each sentence with a sliding window of length h that has a set of m filters applied to it. A feature ci is produced when these filters are applied to each window of words that could possibly exist in the phrase. Every filter has a unique bias of its own. Several feature maps are produced by these m filters operating in concurrently.

Global Max Pooling Layer: The local optimal features and feature map produced by convolution layer are sampled by pooling layer. By combining the data, this layer lessens the representation.

Fully Connected Layer: The Eq. (3), where α is rectified linear unit (ReLU) activation function, W ∈ Rm×m is weight matrix, b ∈ Rm is bias, Cpool is feature map matrix produced by pooling layer, is used by fully connected layer to compute transformation.

Sentence embedding for every review is represented by output vector of this layer. Ultimately, a completely connected softmax layer receives output from preceding layer. The class K with the highest probability is returned. Since error probability between network prediction as well as actual output label is measured in three different classes, “categorical_crossentropy” loss function is employed in the softmax layer. After softmax layer gives classification result, back-propagation method updates the model parameters based on the training data’s actual classification label. Ultimately, three labels with values are assigned to each sentence, one of which corresponds to the actual label. As an illustration, “positive” is equal to [0, 0, 1], “negative” to [1, 0, 0], and “neutral” to [0, 1, 0].

### Feed-Forward convolutional Temporal neural network (FCTNN)

We first explain a generic design for the network’s essential component, convolutional sequence prediction, before specifying the network’s structure^7^. Assume we have a set of malicious userdata {x0,., xT } and we utilise these to forecast the malicious userdata {y0,., yT } at the following period time. To using the current observed data {x0,., xt − 1} as inputs in order to forecast the outputs yt for a certain time t. The following mapping can be defined by the function f: XT → Y T, which represents a sequence modelling network^25,53^.

To forecast the malicious user yt at time t, we have the function f if it meets causal constraint that yt depends only on {x0,…, xt − 1} rather than any “future”’ inputs {xt + 1,…, xT } by Eq. (10)

10 $$\:{\stackrel{\prime }{y}}_{t}=f\left({x}_{0},\dots\:,{x}_{t-1}\right)$$

Finding a network f that can minimise expected loss between actual data and prediction, or L (yˆt, f(x0,., xt − 1)), is the aim of learning the sequence modelling configuration. Suggested deep learning system is feed-forward convolutional temporal neural network (FCTNN). It was initially created for action segmentation and detection^26^, and this is where our FCTNN model receives inspiration and adaptations from. The FCTNN stands out from other neural networks that are currently in use for short-term harmful user forecasting. This trait logically matches the sequence prediction mentioned above^14,27^. At time t, the causal convolutions essentially function as a filter that can only view inputs that are received no later than t. This prevents knowledge from leaking from the past to the present. This enables our framework to process input of any length for a data sequence^28^. It requires extensive networks with a lengthy effective history. This is certain to result in a convoluted network architecture and significant processing overhead^29^. Alternatively, the suggested FCTNN architecture incorporates residual layers and dilated convolutions. Dilated convolutions in particular allow for an increasingly wide receptive field by Eq. (11)

11 $$\:\:F\left(t\right)=\left(x{\text{*}}_{d}f\right)\left(t\right)={\sum\:}_{i=0}^{k-1}\:f\left(i\right)+{x}_{i-d}$$

where t − d · i denotes direction of past, d is dilation factor, and k is the filter size. Every two consecutive filter taps, there is a fixed step that represents the dilation factor. In reality, a regular convolution is a dilated convolution with a dilation factor of d = 1. Dilation factor modifies the TCN’s receptive field. Our methodology ensures an extraordinarily broad effective history by adjusting d exponentially with network depth. As a result, the receptive field can expand due to the increase in dilatation^30^. As a result, a larger range of inputs are represented in the output at the top level. Keep in mind that you may also change the filter size k to expand the FCTNN’s receptive field.

We employed the rectified linear unit (ReLU) for each of the two weight layers in the FCTNN. Furthermore, a spatial dropout for regularisation is included following the final weight layer. Formally, the residual block is defined as follows in this paper by Eq. (12)

12 $$\:y=\mathcal{F}\left(x,{W}_{i}\right)+x$$

Y represents the layer’s output vector in this instance. Two layers are represented by the formula F = W2σ(W1x) + e, where σ stands for ReLU and e for bias. The FCTNN architecture for our framework is built based on the original FCTNN setup, as described in this paper. A set of blocks, each containing a succession of L convolutional layers, composites it. Dilated convolutions, which are connected to a non-linear activation f and a dilation factor d, combine each layer (.). In addition, each dilated convolution has a residual link added to it in order to integrate the layer’s input with the convolution result. Assume that S (i, j) ∈ R Fw×T represents activations for ith layer and jth block. Observe that each layer I has the same number of filters (Fw). We can use processed training data to train TCN method once it has been constructed. The central server will receive the final TCN with optimised structure, which will identify malevolent users.

### Document management using role based access with simulated remora swarm optimization (SRSO)

In private blockchains, smart contracts offer a potent means of implementing role-based access control. They are a crucial part of any reliable and safe blockchain system because they offer transparency, automation^31,32^, and flexibility in the management of access control regulations. Smart contracts can be utilised on a private blockchain to implement RBAC by specifying the roles and permissions of individual users^33^. These roles can be used to limit access to specific system data or operations^34,54^. It is possible to construct the smart contract so that only users who are assigned proper roles can access or carry out particular operations on the contract.

Free travel (Exploration): The global search is carried out by the SRSO using the Sailed Fish Optimizer (SFO) approach, which is based on the elite method employed in the swordfish algorithm. The following is an expression for the position updating formula by Eq. (13)

13 $$\:{V}_{i}(t+1)={X}_{\text{best}}\left(t\right)-\left(\text{r}\text{a}\text{n}\text{d}\times\:\left(\frac{{X}_{\text{best}}\left(t\right)+{X}_{\text{rand}}\left(t\right)}{2}\right)-{X}_{\text{rand}}\left(t\right)\right)$$

And the ith remora’s candidate position is represented by Vi(t + 1). The best position as of right now is Xbest(t). Remora’s random position is denoted by Xrand(t). Iteration number t is what we’re talking about. A random number between 0 and 1 is called a rand. Furthermore, remora has the ability to switch hosts based on its experiences. In this instance, a fresh candidate position by Eq. (14)

14 $$\:{V}_{i}^{{\prime\:}}(t+1)={V}_{i}(t+1)+\text{r}\text{a}\text{n}\text{d}\text{n}\times\:\left({V}_{i}(t+1)-{X}_{i}\left(t\right)\right)$$

SFO Strategy: It is evident that the remora will move with the sailfish once it has adsorbed on it. With the use of the SFO algorithm’s elite approach, the formula is enhanced and yields the following formula by Eq. (15)

15 $$\:{X}_{i}^{t+1}={X}_{\text{Best}}^{t}-\left(\text{r}\text{a}\text{n}\text{d}\:\right.\:\left.\times\:\left(\frac{{X}_{\text{Best}}^{t}+{X}_{\text{rand}}{\:}^{t}}{2}\right)-{X}_{\text{rand}}{\:}^{t}\right)$$

where XBest t is current best position, Xrand t is current random position of Remora, t is iteration number.

Experience Attack: After being adsorbed on the host, the remora will explore a small portion of the host by using the locations of previous and present generations of remora to determine whether host needs to be replaced. This method is similar to experience-building process. Formula for a mathematical calculation is shown in Eq. (16)

16 $$\:Xatt={X}_{i}^{t}+({X}_{i}^{t}+{X}_{pre})\times\:randn$$

where Xatt represents the remora’s tentative movement. As an experience, Xpre might be thought of as the position of the preceding generation of Remora. Lastly, a random number with a normal distribution between 0 and 1 is called rand. The remora uses Eq. (17) to determine whether to switch hosts after a brief range of motion and defines the decision method.

$$\:f\left({X}_{i}^{t}\right)<f\left({X}_{att}\right)$$

17 $$\:H\left(i\right)=\text{r}\text{o}\text{u}\text{n}\text{d}\left(\:\text{r}\text{a}\text{n}\text{d}\:\right)$$

H(i), among them, has an initial value of 0 or 1 and indicates the host absorbed by remora. Whale is absorbed if H(i) equals 1, sailfish is adsorbed if H(i) equals 0. Additionally, round is a rounded function, and the fitness values of Xi t and Xatt are, respectively, f(Xi t) and f(Xatt).

## Experimental analysis

Every experiment in this research was carried out using MATLAB R2021a on a Windows 11 PC with an Intel (R) Core (TM) i7-11700 CPU operating at 2.50 GHz and 16 GB of RAM.

### Dataset description

CERT Dataset: We utilised the “CERT Insider Threat Tools” dataset (Carnegie Mellon’s Software Engineering Institute, Pittsburgh, PA, USA) since it is quite difficult to obtain actual business system logs. CERT dataset is an intentionally manufactured dataset utilised to validate insider-threat detection systems; it is not real-world^35,36^ corporate data. Employee computer usage logs and specific organisational data like employee departments and roles are included in the CERT dataset. Every table has data about each user’s activities, timestamps, and ID. The CERT dataset is available in six significant versions (R1 through R6), with R6.1 and R6.2 being the most recent releases. Depending on the version of the dataset, there are differences in the kinds of usage data^37^, the number of variables, the number of employees, and the quantity of malicious insider activities. The largest and most recent dataset, R6.2, was used for this investigation—only five of the 4000 users in this version of the dataset engaged in harmful behaviour. Table 1 describes the logon activity table.

**Table 1:** Log records of Logon activities.

Recorded Item	Description
User	User ID
Date	Day/Month/Year
PC	Key number of a PC logged on
ID	Primary key of an observation
Activity	Log on or log off (Binary type)

User Activity: This research aims to monitor student behaviour throughout the learning process to assess elements that might affect a student’s academic achievement. There are 150 student records with 11 attributes in the gathered data set. Three primary categories classify the features: (1) Gender and nationality are examples of demographic features. (2) Academic background elements, including section, grade, and stage. (3) Behavioural elements include raising one’s hand during class, accessing resources, participating in discussion groups, and paying attention to messages and announcements. One of the key areas of research in educational psychology is student involvement. “The quality and quantity of students’ psychological, cognitive, emotional, and behavioural reactions to the learning process as well as to in-class/out-of-class academic and social activities to achieve successful learning outcomes” is the definition given to student engagement. Attributes and features of the dataset are displayed in Table 2, along with a description. A behavioural factor is a new feature category visible in the table. These qualities have to do with the experiences that students have and how they behave when they are in school. We use a few preprocessing methods to improve the data set’s quality after the data collection activity. Data preprocessing, which encompasses data transformation, data reduction, data cleansing, and feature selection, is a crucial phase in knowledge discovery.

**Table 2:** Student features and their description.

Feature category	Feature	Description
Demographical features	Nationality	Student nationality
	Gender	Student gender
	Place of birth	Birth place of student
	Relation	Students contact parent such as father or mum
Academic background features	Grade ID	Grade student belongs such as 1,2,3
	Stage ID	Stage student belongs such as lower, middle, high
	Section ID	A, B, C section of student
	Semester	1 st or 2nd semester
	Topic	Course topic such as math, English, Arabic, science
	Teacher ID	Teacher who teach this particular course
Behavioural features	Raised hand on class	How students behave when using the Kalboard 360 e-learning platform
	Viewing announcements	
	Discussion groups	
	Opening resources	

We looked into the roles of the 73 anomalous events, as indicated in Table 3, to determine traits of malevolent insiders. It was discovered that the three roles of “salesman,” “information technology (IT) administrator,” and “electrical engineer” account for almost 90% of the most aberrant actions. Constructing an effective detection model without any abnormal instances in a role is challenging. However, it is also impossible to validate the performance of the produced method in case of roles with fewer than three abnormal instances. The frequency of normal and abnormal instances in 3 roles are shown in Table 3.

**Table 3:** Number of anomalous records according to role.

Role	Number of Anomalous Records
Electrical Engineer	10
IT Admin	23
Salesman	32
Computer Programmer	3
Director	1
Manager	2
Production line worker	1
Software developer	1
Total	73

**Table 4:** Comparative analysis based on dataset feature using proposed and existing technique.

Classifiers	Feature class	Parameters			
		Prediction accuracy	Mean average precision (MAP)	F-1 score	Latency
**CNN**	**Demographic features**	68	72	63	71
	**Academic background features**	73	76	67	74
	**Behavioral factors**	75	79	71	77
**KNN**	**Demographic features**	75	82	74	67
	**Academic background features**	77	84	77	73
	**Behavioral factors**	80	87	80	77
**SVM**	**Demographic features**	82	80	83	80
	**Academic background features**	85	83	85	82
	**Behavioral factors**	87	88	89	85
**Random forest**	**Demographic features**	81	82	84	83
	**Academic background features**	83	84	88	85
	**Behavioral factors**	85	86	90	87
**Proposed B-FCTNN_SRSO** **model**	**Demographic features**	89	88	93	92
	**Academic background features**	94	91	95	94
	**Behavioral factors**	98	95	97	96

**Fig. 6:**
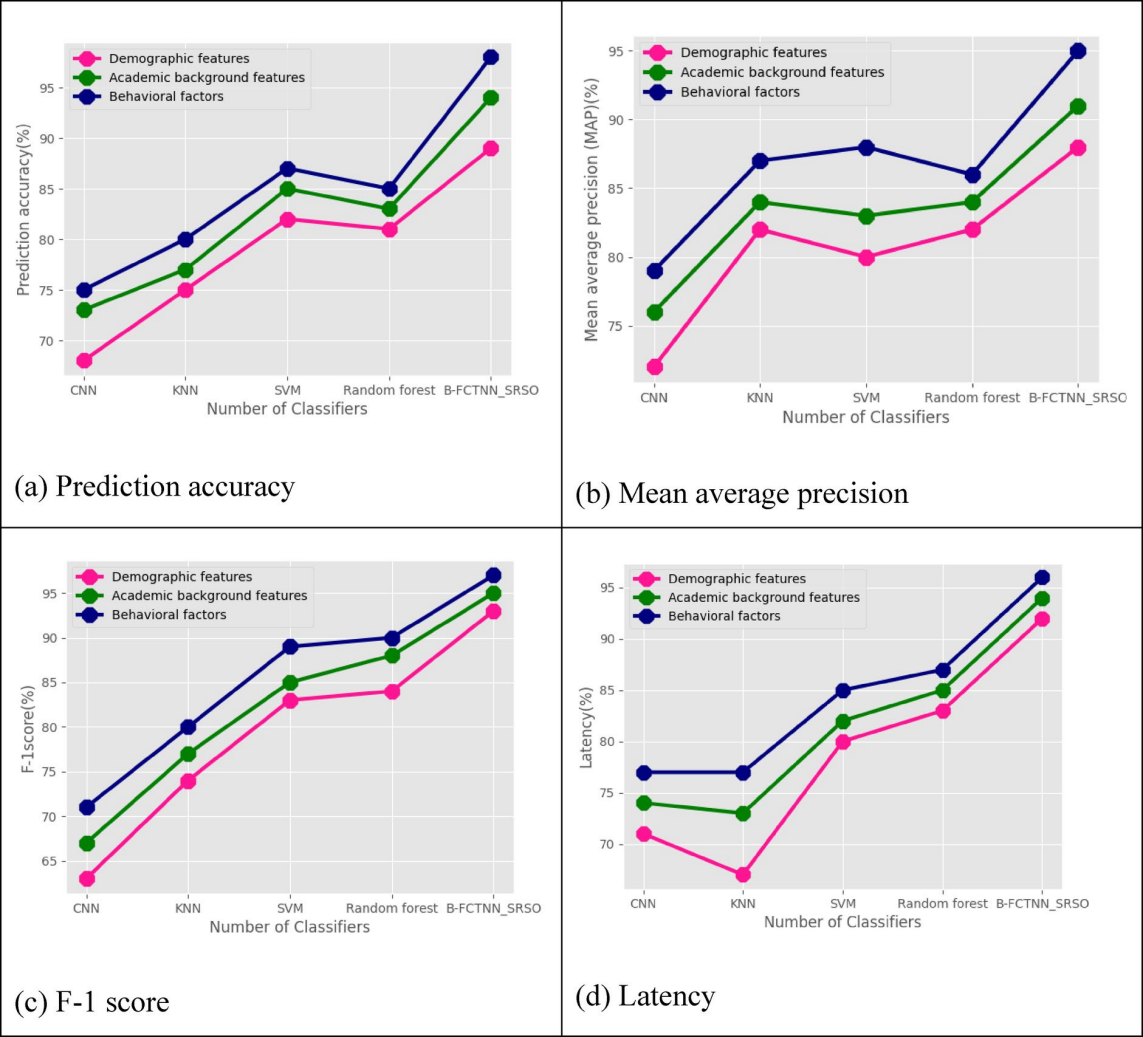
Comparative analysis based on feature class for various classifiers in terms of (**a**) Prediction accuracy, (**b**) MAP, (**c**) F-1 score, (**d**) Latency

 Table 4 and Figure6(a)-(d) compare a classifier based on various feature classes of input student educational data. Here, the feature classes^39^ analyzed are demographic features, academic background features, behavioral factors in prediction accuracy, MAP, F-1 score, And latency. The proposed technique achieved a prediction accuracy of 98%, a mean average precision (MAP) of 95%, an F1 score of 97%, And a latency of 96% for demographic features. For academic background features, it attained a prediction accuracy of 94%, a MAP of 91%, an F1 score of 95%, And a latency of 94%. Regarding behavioural factors, the proposed technique achieved a prediction accuracy of 89%, a MAP of 88%, an F1 score of 93%, And a latency of 92%.

In contrast, existing classifiers—CNN, KNN, SVM, And random Forest—obtained a prediction accuracy of 68%, a MAP of 72%, an F1 score of 63%, And a latency of 71% for demographic features. For academic background features, these classifiers achieved a prediction accuracy of 73%, a MAP of 76%, an F1 score of 67%, And a latency of 74%. For behavioural factors, they attained a prediction accuracy of 75%, a MAP of 73%, an F1 score of 71%, And a latency of 77%.

**Fig. 7:**
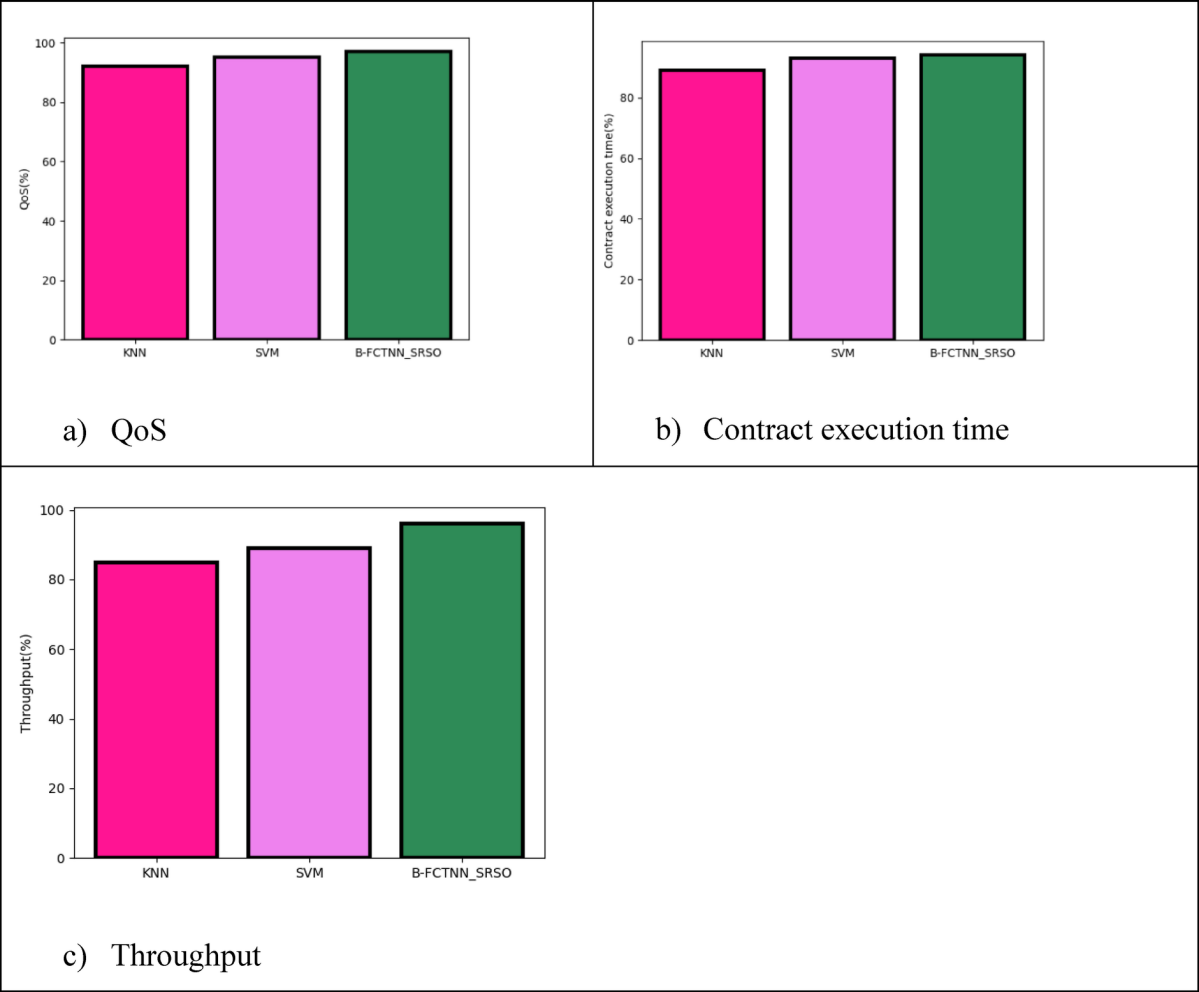
Comparative analysis based on blockchain security analysis in terms of (**a**) QoS, (**b**) Contract execution time, (**c**) Throughput

**Table 5:** Comparative based on blockchain security analysis.

Techniques	QoS	Contract execution time	Throughput
**KNN**	92	89	85
**SVM**	95	93	89
**B-FCTNN_SRSO**	97	94	96

As shown in Table 5, the proposed B-FCTNN_SRSO achieved a QoS of 97%, a precision of 94%, and a throughput of 96% based on blockchain security analysis, as illustrated in Fig. 7(a)-(c). In comparison, the existing KNN technique attained a QoS of 92%, a contract execution time of 89%, and a throughput of 85%, while SVM achieved a QoS of 95%, a contract execution time of 93%, and a throughput of 89%.

**Table 6:** The performance comparison of training, testing, validation accuracy and performance stability with various models.

Model	Training Accuracy (%)	Validation Accuracy (%)	Testing Accuracy (%)	Performance Stability (%)
CNN	80	76	74	75
KNN	85	82	79	82
SVM	90	88	85	87
Random Forest	92	89	87	89
Proposed B-FCTNN_SRSO	98	96	95	96

**Fig. 8:**
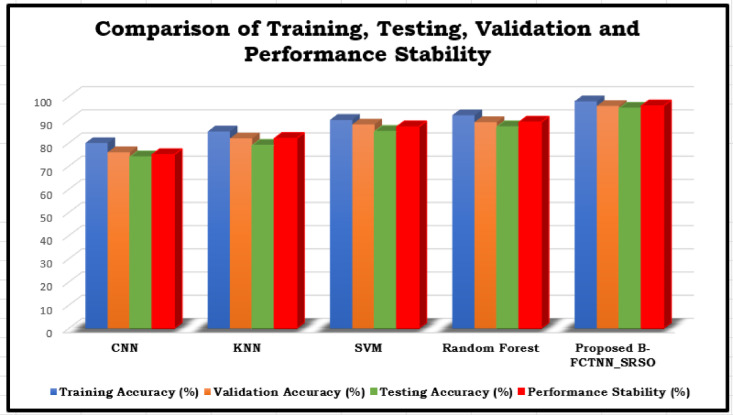
The Performance comparison of Training, Testing, Validation Accuracy and Performance Stability with various models.

Table 6 compares various machine learning models based on their performance stability across dataset splits and their training, validation, and testing accuracy. Overfitting^40,41^ occurs when a model performs well on training data but poorly on unseen data. As shown in Fig. 8, CNN has the lowest testing accuracy at 74%. KNN improves upon this with a validation accuracy of 82% and a testing accuracy of 79%, though its stability remains 82%, indicating minor discrepancies across dataset splits^42^. SVM further enhances testing accuracy to 85%, but inconsistencies between training and validation suggest challenges with generalization^43^. Random Forest performs well, achieving an 87% testing accuracy and 89% stability. The proposed B-FCTNN_SRSO model outperforms all others, achieving the highest stability (96%), training accuracy (98%), validation accuracy (96%), and testing accuracy (95%).

**Fig. 9:**
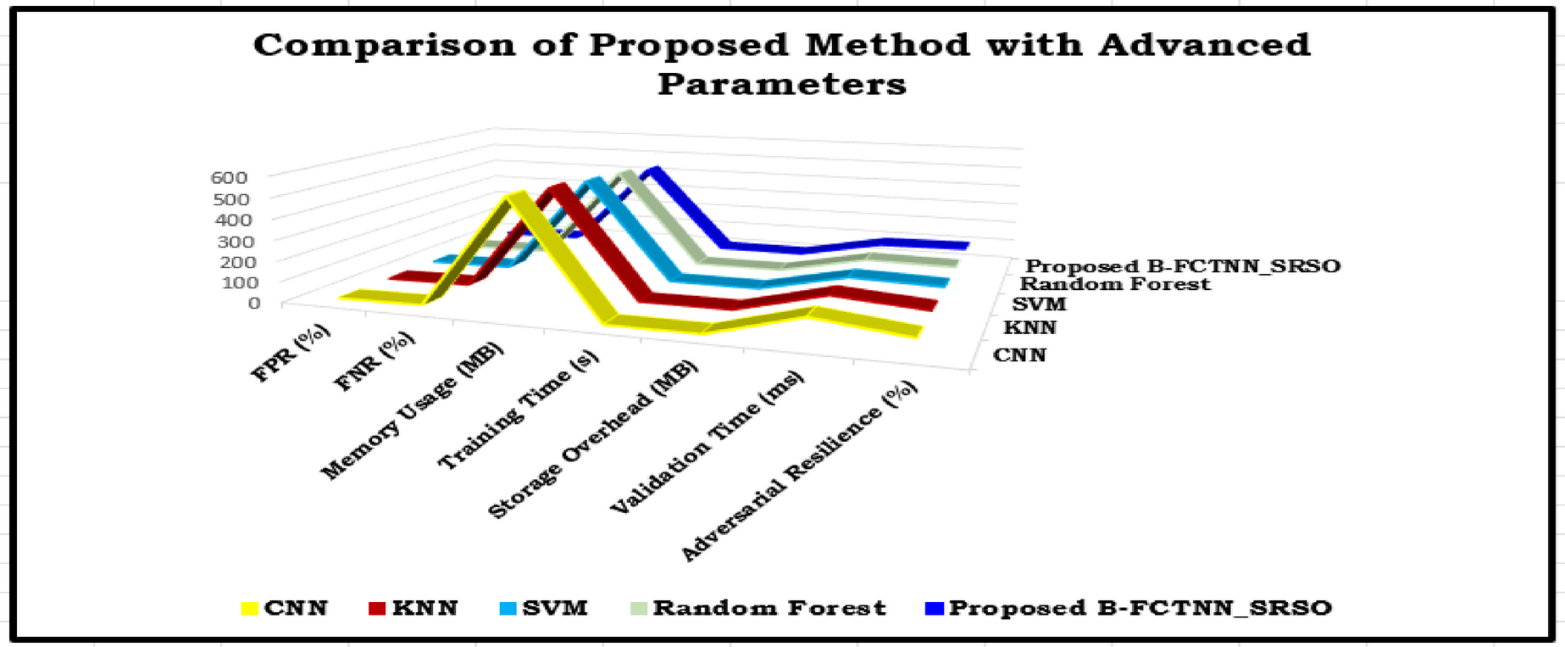
The comparison of proposed method thru advanced parameters against state of art methods.

Table 7 highlights the proposed B-FCTNN_SRSO model, comparing various techniques based on multiple performance metrics. False positive rates (FPR) and false negative rates (FNR) are key indicators of detecting malicious users effectively. As shown in Fig. 9, the proposed model achieves a significantly lower FPR (2%) and FNR (5%) compared to CNN (FPR: 10%, FNR: 15%), demonstrating superior accuracy in threat detection. Regarding memory efficiency, the B-FCTNN_SRSO model outperforms CNN (550 MB) and KNN (520 MB), utilizing only 430 MB of memory. Its drastically reduced training time of 1.2 s makes it highly suitable for real-time applications^43,44^. Additionally, it enhances blockchain system efficiency by minimizing storage overhead to just 0.8 MB and reducing validation time to 85 ms. The proposed model maintains lightweight data^45^ handling compared to CNN (1.8 MB) due to its lower storage requirements. Furthermore, the B-FCTNN_SRSO model offers the highest adversarial resistance (92%), making it more resilient to cyber threats. This upgrade significantly enhances the security of blockchain-based document management, surpassing CNN (72%) and KNN (78%). Overall, the proposed model delivers superior accuracy, efficiency^46^, speed, and security, making it the most effective solution for managing educational documents electronically.

**Table 7:** Comparative analysis of proposed B-FCTNN_SRSO method with some advanced parameters.

Method	FPR (%)	FNR (%)	Memory Usage (MB)	Training Time (s)	Storage Overhead (MB)	Validation Time (ms)	Adversarial Resilience (%)
CNN	10	15	550	2.5	1.8	120	72
KNN	9	12	520	2.1	1.5	110	78
SVM	6	10	490	1.8	1.2	95	83
Random Forest	5	9	470	1.6	1.1	90	85
Proposed B-FCTNN_SRSO	2	5	430	1.2	0.8	85	92

**Table 8:** Comparative analysis of proposed B-FCTNN_SRSO method with existing methods.

Method	Computational Complexity	Security & Robustness	Scalability	Blockchain Integration Efficiency
GANs	High	Moderate	Moderate	Limited
RNNs	High	Moderate	Low	Limited
Hybrid Federated Learning	High	High	High	Moderate
Proposed B-FCTNN_SRSO	Low	High (Blockchain-secured authentication)	High	Highly efficient (Smart contract-based access control)

Table 8 compares different methods based on computational complexity, security, scalability, and blockchain integration efficiency. The model GANs and RNNs have high computational complexity^47^ and limited blockchain integration, making them less efficient for secure document management. Hybrid Federated Learning provides high security and scalability but requires significant computational resources^48^. In contrast, the proposed B-FCTNN_SRSO offers low computational complexity, high security through blockchain authentication, and excellent scalability^49^. Its smart contract-based access control ensures efficient blockchain integration, making it more suitable for secure and scalable electronic document management^50^.

**Table 9:** The comparative analysis of existing optimization algorithms.

Algorithm	Convergence Speed	Computational Complexity	Optimization Efficiency	Scalability	Security in Access Control
PSO	Moderate	High	Moderate	High	Limited (Sensitive to parameter tuning)
Genetic Algorithm	Slow	Very High	High	Moderate	Moderate (Prone to premature convergence)
SRSO (Proposed)	Fast	Low	High	High	High (Dynamic adaptation to role-based access)

Table 9 compares PSO, Genetic Algorithm, and the SRSO proposed regarding convergence speed, computational complexity, optimization efficiency, scalability^50^, and access control security. The PSO offers average convergence speed but consumes high computational power and optimized parameters, thus making it less efficient for access control^51^. Genetic Algorithms offer high optimization efficiency but slow convergence speed and high computational complexity, thus prone to premature convergence. SRSO, however, is the fastest, offers low computational complexity, and is highly scalable and secure^52^. Its dynamic adaptation of role-based access control makes it the most efficient solution for secure blockchain applications.

**Table 10:** The security analysis of various algorithms.

Algorithm	Sybil Attack Resistance (%)	Smart Contract Security (%)	Privacy Protection (%)	Scalability in Blockchain (%)	Overall Security Efficiency (%)
PSO	50	60	55	65	58
Genetic Algorithm	65	65	50	55	59
Federated Learning	85	50	90	85	78
Proposed SRSO-Based Model	95	90	95	92	93

**Fig. 10:**
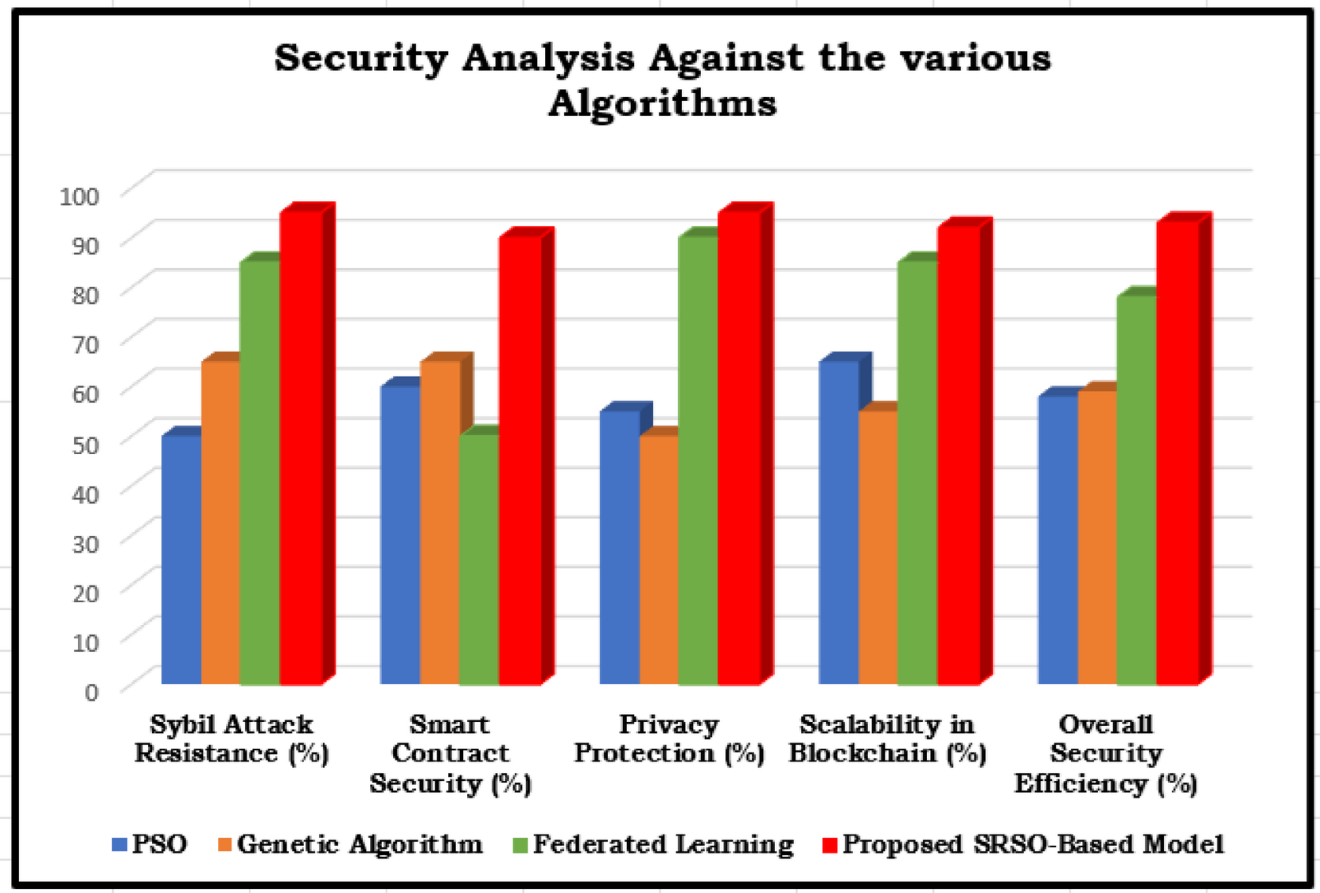
The Security Analysis against the Various Models.

Table 10 compares various algorithms regarding blockchain security risks, including Sybil attack resistance, privacy protection, scalability, and overall security efficiency.

PSO and Genetic Algorithms perform poorly securing blockchain-based systems, exhibiting only moderate smart contract security and weak Sybil attack resistance (50–65%). Additionally, privacy protection remains inadequate (50–55%), posing a significant risk of data leakage. In contrast, Federated Learning offers strong privacy protection (90%) and scalability (85%), ensuring better data security. However, its weak smart contract security (50%) leaves it vulnerable to contract exploits, reducing its reliability. As illustrated in Fig. 10, the proposed SRSO-Based Model outperforms all other approaches, achieving 95% Sybil attack resistance, 90% smart contract security, and 95% privacy protection. Its high scalability (92%) ensures effective role-based access control in blockchain-based educational document management. Regarding enhancing security and efficiency in blockchain applications, SRSO stands out as the most scalable and secure solution.

### Comparative analysis against the existing blockchain based credential verification systems

**Table 11:** Comparative analysis against the existing blockchain based credential verification systems.

Method	Accuracy (%)	Verification Speed (ms)	Scalability (%)	Security Efficiency (%)	Resistance to Attacks (%)	Smart Contract Optimization (%)
Hyperledger Fabric-Based System ^22^	85	120	80	82	75	78
Ethereum Smart Contract System ^23^	88	110	85	85	78	80
Decentralized Identity (DID) System ^24^	90	100	88	87	82	85
Proposed B-FCTNN_SRSO	98	85	95	96	94	92

**Fig. 11:**
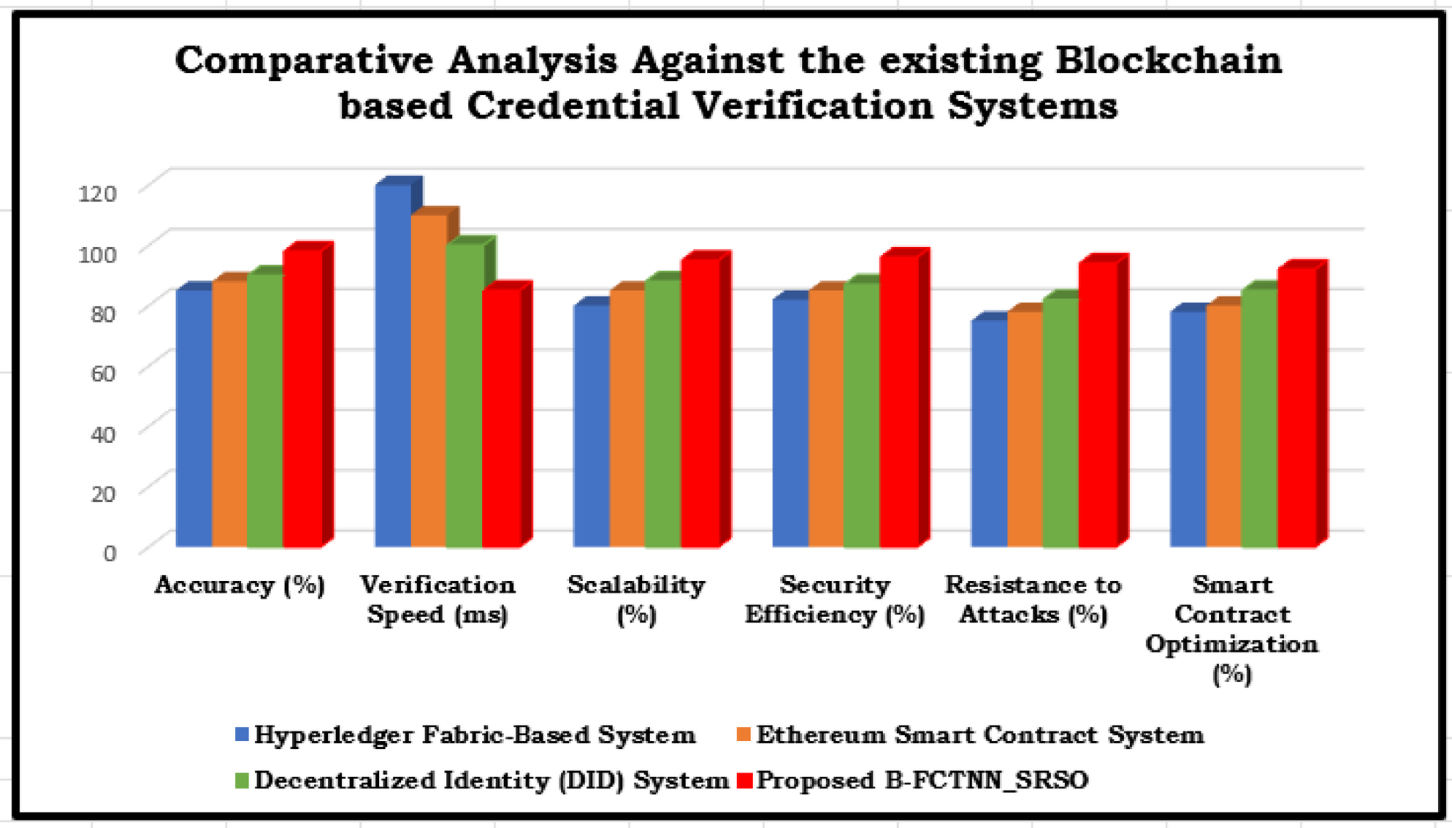
Comparative Analysis Against the existing Blockchain based Credential Verification Systems.

As per Fig. 11, a comparative analysis is performed against the existing Blockchain based credential verification systems. Table 11 compares various blockchain-based credential verification systems based on efficiency, security, speed, attack resistance, accuracy, and smart contract optimization. The Hyperledger Fabric-Based System is less efficient for large-scale applications due to its slower verification time (120 ms) and lower accuracy (85%). The Ethereum Smart Contract System improves verification speed to 110 ms and accuracy to 88%, but its attack resistance remains relatively low at 78%. While the Decentralized Identity (DID) System further enhances security (87%) and attack resilience (82%), its verification speed remains moderate at 100 ms.As illustrated in Fig. 11, the proposed B-FCTNN_SRSO technique outperforms all competing methods, achieving higher accuracy (98%), faster verification speed (85 ms), improved scalability (95%), and superior security efficiency (96%).

## Discussion

A system that uses machine learning algorithms to analyse and improve the management of educational documents, such as transcripts, student records, and certificates, stored safely on a blockchain network. This system offers features like automated verification, personalised learning insights, and fraud detection, all while preserving data integrity and transparency throughout the educational ecosystem.

- Scalability: It cannot be easy to handle massive amounts of educational data on a blockchain, mainly when dealing with intricate learning algorithms.
- Adoption Barriers: It might be necessary to make considerable adjustments to infrastructure and protocols to incorporate blockchain technology into current educational systems.
- Privacy Issues: The advantages of blockchain transparency must be carefully weighed against data privacy.

## Conclusion

The novel method for managing educational documents was proposed through a blockchain-based fuzzy feed-forward convolutional temporal neural network to detect malicious users. This instance uses NLP analysis to document word weight indexing during training. The role-based access control combined with simulated remora swarm optimization enforces document management access control. The suggested architecture aims to identify rogue users by authenticating users and logging access requests on the blockchain. This may be the first study to integrate student behavior with academic success. The high accuracy results validate the newly acquired information from categorization approaches, which finds that learners’ activities played a significant role in the learning process. Based on user behavior modelling and anomaly detection methods, we suggested an insider-threat detection framework. Individual users’ diverse behaviors are converted into a structured dataset throughout the user behavior modelling process, where each row corresponds to an instance, and every column corresponds to input variables for anomaly detection methods. We identified future research directions due to the current study’s limitations, even though the proposed framework was empirically verified. Our insider-threat detection model was constructed using a specific time unit, such as a day. In another way, this method can identify harmful activities based on batch process but cannot instantly identify them. Therefore, creating an online stream data-processing sequence-based insider-threat detection method could be worthwhile. In order to obtain more accurate results, our future work will involve applying data mining algorithms on an expanded data set with additional distinguishing qualities.

## Data Availability

The datasets used and/or analysed during the current study available from the corresponding author on reasonable request.
